# Peer Review of “Use of Smartphone Apps for Improving Physical Function Capacity in Cardiac Patient Rehabilitation: Systematic Review”

**DOI:** 10.2196/33181

**Published:** 2021-09-17

**Authors:** Karla Goessler

**Affiliations:** 1 Faculty of Medicine University of Sao Paulo Sao Paulo Brazil

**Keywords:** cardiac rehabilitation, physical capacity, exercise, smartphone apps

*This is a peer-review report submitted for the paper “Use of Smartphone Apps for Improving Physical Function Capacity in Cardiac Patient Rehabilitation: Systematic Review”.*

## Review Round 1

### General Comments

This is a systematic review [1] investigating the utilization of smartphone apps for improving physical function capacity in cardiac rehabilitation (CR). Please find below my comments/suggestions.

### Specific Comments

#### Major Comments

1. CR interventions seem to be quite different between studies, making future comparisons inappropriate (ie, for CR programs including exercise programs, I would expect improvements in cardiorespiratory fitness (CRF), while for programs including diet, this outcome might not change).

2. It is not clear how authors selected the papers. This process makes it difficult to understand the results, as the outcomes and types of interventions are quite different between studies. As the main outcome in CR is CRF, I would suggest making it your primary outcome for selection of the studies.

#### Minor Comments

##### Methods/Results

1. I would suggest including a PRISMA (Preferred Reporting Items for Systematic Reviews and Meta-Analyses) flow diagram for the study selection process. This is available at http://prisma-statement.org/prismastatement/flowdiagram.aspx.

2. Tables 2 and 3 are not clear.

## Abbreviations

CR: cardiac rehabilitation
CRF: cardiorespiratory fitness
PRISMA: Preferred Reporting Items for Systematic Reviews and Meta-Analyses
